# Seminal shedding of human herpesviruses

**DOI:** 10.1186/1743-422X-10-226

**Published:** 2013-07-08

**Authors:** Maja D Kaspersen, Per Höllsberg

**Affiliations:** 1 Department of Biomedicine, Aarhus University, Aarhus C DK-8000, Denmark

**Keywords:** Herpesviruses, HSV, EBV, HCMV, HHV-6A/B, HHV-7, Sperm, Semen, Sexual transmission, Fertility

## Abstract

Most of the human herpesviruses can be found in semen, although the reported prevalence varies considerably between individual studies. The frequent presence of herpesvirus in semen raises the question whether sexual transmission of the virus could have an impact on human reproduction. Only few studies have associated seminal shedding of herpesviruses with impaired sperm quality, reduced fertility, or reduced chances of pregnancy, whereas most studies fail to find an association. Taken together, no firm evidence is so far linking the presence of herpesviruses in semen to impaired human reproduction.

## Introduction

It is well established that many pathogens may cause sexually transmitted diseases (STD). Examples include bacterial infections with *Chlamydia trachomatis, Neisseria gonorrhoeae, and Treponema pallidum*, which may impair fertility or cause severe congenital disorders. Also viral infections with herpes simplex virus type 2 (HSV-2), causing genital herpes, or human immunodeficiency virus (HIV), which untreated may lead to AIDS, are significant health care issues. It is generally believed that transmission of pathogens causing STD occurs through direct contact between mucous membranes, however accumulating reports have indicated that several bacteria [1,2] and viruses, including HIV [3], human papillomavirus (HPV) [4], hepatitis B virus [5], hepatitis C virus [6], Ebola virus [7], adenovirus [8], and various human herpesviruses (HHV) are present in semen. This suggests that semen may also be an important transmission route, although its precise role can be difficult to establish. This review will focus on herpesviruses in semen and their potential significance for human reproduction.

The family of *Herpesviridae* consists of nine structurally similar, large, enveloped DNA viruses, which all share the ability to persist latently in the host cell and give rise to recurrent infections. The HHV have been divided into three subfamilies (α, β, and γ) based on both virological and biological properties, including the type of primary target cells and the site of latency. No studies today have examined the individual prevalence of all nine HHV in semen. The individual studies are summarized in Table 1 (α- and γ-HHV) and Table 2 (β-HHV).

**Table 1:** Presence of α- and γ-herpesviruses in human semen

**Study**	**HSV-1/2**	**VZV**	**EBV**	**HHV-8**	**Samples from**
Aynaud et al. 2002 [9]	9% (n = 111)				Male partners of females with genital HPV lesions France
Bagasra et al. 2005 [10]				A) 4.4% (n = 45)	A) HIV negative B) HIV-positive homosexual men USA, Japan
				B) 87.7% (n = 73)	
Bezold et al. 2001 [11]	3.2% (n = 252)	0% (n = 252)	7.1% (n = 252)	0% (n = 252)	FCA Germany
Bezold et al. 2007 [12]	3.7% (n = 241)		0.4% (n = 240)		FCA Massachusetts
Bocharova et al. 2007 [13]	51.7% (n = 29)				FCA Russia
Chen et al. 2013 [14]	25.5% (n = 153)		3.9% (n = 153)		FCA China
El Borai et al. 1997 [15]	A) 24% (n = 153)				A) FCA B) Fathers Japan
	B) 0% (n = 16)				
Howard et al. 1997 [16]				A) 25% (n = 24)	A) HIV-infected men B) Healthy sperm donors UK
				B) 0% (n = 115)	
Kapranos et al. 2003 [17]	49.5% (n = 113)		16.8% (n = 113)		FCA Greece
Kaspersen et al. 2012 [18]	HSV-1: 0.4% (n = 198) HSV-2: 0.1% (n = 198)	0% (n = 198)	6.3% (n = 198)	0% (n = 198)	Sperm donors Denmark
Kelsen et al. 1999 [19]				0% (n = 100)	Sperm donors Denmark
Lisco et al. 2012 [20]	A) 0% (n = 28)		A) 3.5% (n = 28)		Semen plasma from A) HIV-negative men B) HIV-positive men Italy
	B) 8% (n = 50)		B) 56% (n = 50)		
McGowan et al. 1983 [21]	0% (n = 210)				A) FCA B) Healthy students Australia
Michou et al. 2012 [22]	29% (n = 109)	0% (n = 109)	45% (n = 109)		Men from couples undergoing IVF treatment Spain
Neofytou et al. 2008 [23]	2.3% (n = 172)	2.3% (n = 172)	40.6% (n = 172)		FCA Greece
Rinaldo et al. 1992 [24]	0% (n = 116)				HIV-pos. (n = 58) and HIV-neg. (n = 58) homosexuals USA
Rintala et al. 2004 [25]	3.2% (n = 252)				Fathers to be Finland
Rohde et al. 1999 [26]	3.7% (n = 241)				Infertile men Germany
Thomas et al. 2006 [27]			3% (n = 33)		FCA UK
Wald et al. 1999 [28]	47% (n = 15)				Men with genital HSV-2 Washington

**Table 2:** Presence of β-herpesviruses in human semen

**Study**	**HCMV**	**HHV-6A/B**	**HHV-7**	**Samples from**
Aynaud et al. 2002 [9]	6.3% (n = 111)			Male partners of females with genital HPV-lesions France
Bantel-Schaal et al. 1993 [29]	0% (n = 63)			FCA Germany
Bezold et al. 2001 [11]	3.6% (n = 252)	4.0% (n = 252)	0.4% (n = 252)	FCA Germany
Bezold et al. 2007 [12]	8.7% (n = 241)	3.7% (n = 241)		FCA Massachusetts
Bresson et al. 2003 [30]	5.3% (n = 197)			Sperm donors France
Chen et al. 2013 [14]	21.6% (n = 153)	2.0% (n = 153)		FCA China
Diafouka et al. 2007 [31]	14.3% (n = 63)			FCA Ivory Coast
Eggert-Kruse et al. 2009 [32]	6.5% (n = 170)			Subfertile patients Germany
Erles et al. 2001 [8]	A) 14.3% (n = 14)			A) Normal sperm quality (SQ) B) Abnormal SQ Germany
	B) 0% (n = 43)			
Handsfield et al. 1985 [33]	A) 22% (n = 18)			A) HCMV-positive partner B) HCMV-negative partner USA
	B) 0% (n = 42)			
Howard et al. 1997 [16]	A) 83.3% (n = 24)			A) HIV-infected men B) Healthy sperm donors UK
	B) 3.5% (n = 115)			
Kapranos et al. 2003 [17]	7.1% (n = 113)			FCA Greece
Kaspersen et al. 2012 [18]	2.7% (n = 198)	13.5% (n = 198)	4.2% (n = 198)	Sperm donors Denmark
Lang and Kummer 1975 [34]	A) 9.4% (n = 32)			A) University students/personnel B) Patients with venereal infections C) FCA North Carolina
	B) 10% (n = 10)			
	C) 1.1% (n = 185)			
Levy et al. 1997 [35]	2.85% (n = 70)			Only CMV-IgG positive men France
Lisco et al. 2012 [20]	A) 3.5% (n = 28)	A) 6% (n = 28)	A) 6% (n = 28)	Semen plasma from A) HIV-negative men B) HIV-positive men Italy
	B) 70% (n = 50)	B) 2% (n = 50)	B) 12% (n = 50)	
Mansat et al. 1997 [36]	5.1% (n = 97)			Sperm donors France
McGowan et al. 1983 [21]	A) 2.4% (n = 170)			A) FCA B) Healthy students Australia
	B) 2.5% (n = 40)			
Michou et al. 2012 [22]	43% (n = 109)	8.2% (n = 109)	3.6% (n = 109)	Men in couples undergoing IVF treatment Spain
Naumenko et al. 2011 [37]	A) 11% (n = 91)			A) Infertile men B) Fertile men Russia
	B) 15% (n = 47)			
Neofytou et al. 2009 [23]	56.9% (n = 172)	66.8% (n = 172)	0% (n = 172)	FCA Greece
Rinaldo et al. 1992 [24]	A) 33% (n = 58)			A) HIV-pos. homosexuals B) HIV-neg. homosexuals USA
	B) 17% (n = 58)			
Shen et al. 1994 [38]	32.7% (n = 217)			FCA China
Sherertz et al. 1984 [39]	29.4% (n = 17)			Homosexuals USA
Yang et al. 1995 [40]	33.5% (n = 248)			FCA Taiwan

### Herpes simplex virus-1 and -2

HSV-1 and -2 are neurotropic α-HHVs. HSV-1 is primarily associated with oral transmission and recurrent oral lesions, whereas HSV-2 is the major cause of genital herpes, although both viruses are capable of infecting any region of the body. Primary or recurrent infections can occur either symptomatically or asymptomatically. The seroprevalence of HSV-1 and HSV-2 increases with age in both Europe and the USA, reaching a plateau around age 40 with a prevalence of approximately 70% for HSV-1 and 40-60% for HSV-2 in “high-risk” populations and 20-35% for HSV-2 in “low-risk” populations [41]. The prevalence of HSV-1 is roughly similar in Europe and the USA, whereas the prevalence of HSV-2 is considerable higher in the USA [41]. Frequently, studies have not discriminated between HSV-1 and -2; in these cases, we refer to HSV-1/2. HSV-1/2 is sexually transmissible [42], and mother-to-child transmission occurs in 1:1,400-30,000 births, occasionally causing a life threatening generalized infection in the new-born child [43].

The prevalence of HSV-1/2 in semen varies substantially between different studies (Table 1). More than half of the studies have reported frequencies of less than 4% in both fertile and infertile men, but particularly studies from Greece [17] and Russia [13] find, using nested PCR and in situ hybridization, respectively, that almost half of the semen samples from fertility-clinic attendants contain HSV-1/2. Moreover, also using nested PCR a study from Japan [15] found HSV-1/2 in semen from 24% of fertility-clinic attendants, but none in semen from their fathers. Whether these significant variations in prevalence are due to methodological differences, or reflect a true variation between study populations may require additional analyses to solve. Out of four studies, three found the parameters in routine sperm analyses to be normal in sperm from HSV-1/2 positive men or in sperm exposed to HSV-1/2 in vitro [11,23,44]. The fourth study found that the presence of HSV-1/2 was associated with low sperm count and poor motility [17]. Indeed, HSV-2 isolated from the testis of a dead man has been reported infectious [45], and one case of HSV-2 transmission through donor semen to a recipient, who subsequently acquired a primary infection, has also been reported [42]. Moreover, Bocharova et al. [13] have reported that HSV-2 can be internalized into the heads of morphological normal and motile sperm, suggesting a potential effect of HSV on pregnancy and outcome. Other groups have not yet confirmed this interesting observation.

Taken together, the data so far do not suggest that HSV-1/2 is involved in impaired fertility. Besides from a study of transmission by insemination, the mucosa-associated HSV-1/2 transmission makes it difficult to estimate the risk of HSV-1/2 transmission in semen per se. If an association with sperm heads is a general feature of HSV-2, this might have implications for infection of the endometrium, and warrants additional investigations to clarify.

### Varicella-zoster virus

Varicella-zoster virus (VZV) is an α-herpesvirus infecting most unvaccinated people during early childhood. Primary infection manifests itself as a usually benign childhood disease, varicella (chicken pox), whereas reactivation results in zoster (shingles).

Only few studies have investigated the potential presence of VZV in semen. Except for one study from Greece [23], there is agreement that VZV is neither present in semen from fertility-clinic attendants (Germany) [11], nor from men of couples undergoing IVF treatment (Spain) [22], or from healthy sperm donors (Denmark) [18]. Taken together, the evidence indicates that VZV is not present in semen, perhaps reflecting the infrequency of VZV reactivation occurring from the ganglia rather than immune cells.

### Epstein-Barr virus

Epstein-Barr virus (EBV) is a γ-herpesvirus that is primarily B-lymphotropic, although it may also infect and replicate in epithelia cells in vivo. The age of acquisition of EBV (measured by antibodies) varies in different geographic areas. When acquired in early childhood, the infection is usually asymptomatic, whereas primary infection during puberty may cause infectious mononucleosis. The transmission during puberty involves saliva with high-titered EBV, but might also be transmitted through genital secretions, probably through cell-associated EBV [27,46]. EBV can be present in cervical cells [27] and can be transmitted from mother-to-child in approximately 3% of EBV PCR positive mothers [47]. It has not been reported whether the presence of EBV might impair endometrium receptivity.

An association with leukocytospermia and an increased mean sperm count have been reported in EBV-positive semen samples [11,23], whereas others do not find an impact on sperm count or motility [17]. Anyhow, it is not known whether the prevalence of EBV in semen of infertile and fertile men differs. Separate studies on sperm donors and fertility-clinic attendants demonstrate variations in prevalence between studies from 0.4 to 45% (Table 1). A recent study found significantly increased prevalence of EBV in semen and blood of HIV-infected individuals. Even in cases with no detectable EBV in the blood, EBV was present in semen suggesting compartmentalized reactivation [20].

In conclusion, it has not been convincingly demonstrated that EBV in semen impairs sperm function. Estimates indicate that sexual transmission is a minor route of infection, but the demonstration of EBV in cervical cells may warrant further investigation of a potential role of EBV during reproduction.

### Human herpesvirus 8

HHV-8, also known as Kaposi’s Sarcoma-Associated Herpesvirus or KSAV, is a γ-herpesvirus that primarily, but not exclusively, infects B cells. Infection with HHV-8 is predominantly seen in certain geographic regions in Africa and South America, but this is usually among older adults. The infection is also observed in immunosuppressed individuals, but is rare in healthy individuals from North America, North Europe and Asia. Not surprisingly, most studies have therefore failed to detect HHV-8 in sperm from healthy donors or fertility-clinic attendants (Kaspersen et al., unpublished observation and [11,19]). In contrast, Bagasra et al. reported HHV-8 in 2 of 45 semen samples from HIV-negative men, but further demographic information on these men was not provided. However, HHV-8 was found in semen samples from 64 of 73 (88%) HIV-positive homosexual men. HHV-8 in semen was present in both sperm and mononuclear cells [10]. The prevalence of HHV-8 in previous studies have ranged from 0 to more than 90%, which may reflect methodological differences (including sensitivity and PCR contaminations), differences in HIV status, or geographic and population-based differences (see ref [48], and references herein).

### Human cytomegalovirus

HHV-5 or human cytomegalovirus (HCMV) is a β-herpesvirus to which seroconversion occurs throughout life. Infection by HCMV is often asymptomatic, but clinically important infections occur in immunocompromised individuals or in pregnant women that subsequently may give birth to a child with congenital infection. Severe congenital HCMV infection is associated with growth retardation, mental retardation, deafness, microcephaly, hepatosplenomegaly, chorioretinitis, calcification, and neurologic impairment (for review, see Revello and Gerna [49]).

It has been almost four decades since HCMV was first reported in semen from American men of various populations [50]. Since then, numerous reports on identification of HCMV in semen from differently defined population groups have accumulated. The majority of these studies report a prevalence of approximately 6% in men from Germany, North America, France, Africa, Greece, Denmark, Australia, and Russia (Table 1). In contrast, several studies on semen of fertility-clinic attendants from China, Taiwan, Spain, and Greece, have reported high prevalences of HCMV between 21.6% to 56.9% [14,22,23,38,40]. This could possibly be explained by geographic variation, however another study from Greece, also of semen from fertility-clinic attendants, found HCMV in only 7.1% [17]. Both Greek studies used PCR for HCMV DNA detection; the study that detected the lower frequency even used nested PCR [17]. Shedding of HCMV in semen is also relatively high in homosexual men [24,39]. Howard et al. found shedding of HCMV in 20 out of 24 HIV-positive, homosexual men (83.3%), but only in 4 out of 115 healthy donors (3.5%) [16].

Although one case of hematospermia has been associated with HCMV shedding [51], most groups have found the sperm parameters to be unaffected by HCMV [8,11,17,23,31,32,40,44], and two studies that directly compare the prevalence of HCMV in semen from fertile and infertile men find similar frequencies within the two groups [37,50].

HCMV in semen may be infectious [20,24,39], and HCMV has also been isolated from human endometrial cells [52], suggesting a possible mechanism of direct infection of the endometrial cells by HCMV carried by sperm. Moreover, productive HCMV infection can be obtained in human endometrial stroma cells [53]. Since a primary HCMV infection is associated with an increased risk for early abortion [54,55] and congenital defects in the fetus in general [56], HCMV in semen remains a potential risk of viral transmission, even though this may be an infrequent route of infection.

Whether HCMV directly impairs sperm fertility has been investigated by Eggert-Kruse and coworkers [32]. From subfertile couples, semen from 170 males and endocervical material from 156 females were screened for the presence of HCMV by nested PCR. The presence of HCMV was not concordant between couples, emphasizing that sexual transmission of HCMV is not a frequent route of infection. Furthermore, no significant correlations between HCMV and semen quality or between cervical HCMV-infection and mucus quality or female infertility factor were seen.

In conclusion, sexual transmission of HCMV is rare, but semen may contain infectious virus with the potential of initiating a primary infection. However, there is no evidence that HCMV in semen impairs fertility [32]. Longitudinal studies on pregnancy outcome in HCMV-positive versus HCMV-negative patients are, however, lacking.

### Human herpesvirus 6A/B

Closely related to HCMV, the β-herpesviruses HHV-6A and HHV-6B are frequently examined by methods that do not allow a distinction between the two viruses. Infection usually occurs in the first years of life giving rise to the childhood disease exanthem subitum, and most adults in the Western world are seropositive. HHV-6B has tropism for mononuclear cells, primarily T cells, but is usually found in saliva, which is thought to be the major route of transmission.

Only few reports are available on the prevalence of HHV-6A/B in human semen. Bezold et al. found the prevalence to be 4.0% in a German population of infertility patients [11] and 3.7% in a population of American infertility patients [12]. Chen et al. found HHV-6A/B in 2.0% of infertility patients from China [14], whereas Michou et al. detected HHV-6A/B in 8.2% of 109 men from couples undergoing IVF treatment in Spain [22]. A slightly higher prevalence of 13.5% was seen by Kaspersen et al. in semen from Danish sperm donors [18] (Table 2), suggesting that the prevalence of HHV-6A/B is not increased among patients attending a fertility clinic. A remarkably high prevalence of 66.8% among men attending a fertility clinic on Crete has been reported by Neofytou et al. using nested PCR [23]. The discrepancies in HHV-6A/B prevalence are most likely not due to geographic or population-based differences, since HHV-6A/B appears to be present in all the populations. The most likely explanations are therefore that the nested PCR procedure of Neofytou et al. might be more sensitive or was contaminated. Nonetheless, it is clear that HHV-6A/B does not directly affect sperm parameters [11,23]. Interestingly, HHV-6A/B is associated with the acrosome of the sperm [18], but it is not known whether this provide a mechanism for infecting the endometrium.

The binding of HHV-6B to the acrosome [18] suggests that HHV-6B may be transmitted to the uterus by the sperm, but at the same time this may argue against infection of the oocyte during normal fertilization, since the acrosome is dissolved prior to the sperm enters the egg. Nevertheless, HHV-6A/B integrates chromosomally at a frequency of 0.8% [57], and it remains unknown how this integration might happen. Thus, it is expected that PCR-based detection should identify approximately 1% of semen samples simply due to chromosomal integration of either HHV-6A or HHV-6B.

In conclusion, there is no evidence so far to indicate that HHV-6A/B affects sperm function or other aspects of human reproduction, although a potential role of HHV-6A/B on the endometrium has not been resolved yet.

### Human herpesvirus 7

HHV-7 is a β-herpesvirus closely related to HHV-6A and HHV-6B. HHV-7 is usually acquired prior to age 5, and seroprevalence in adults reaches 96% [58]. Infection by HHV-7 is either asymptomatic or causes exanthem subitum.

Similar to HHV-6A/B, the prevalence of HHV-7 in semen is also based on only few publications. The prevalence in semen from European sperm donors or men attending a fertility clinic is similar and within the range of 0.4 to 6.0% (Table 2). HIV-positive men have a slightly higher prevalence, which could be explained by reactivation of HHV-7 by HIV, although the increase in HHV-7 prevalence is not nearly at the same level of that seen for HCMV [20]. HHV-7 has been detected in a small percentage of analyzed cervical swaps [59] and in one out of eleven analyzed placental biopsy samples [60], but any association to human reproduction has not been reported. Effects of HHV-7 on sperm parameters have not been evaluated.

In conclusion, there is no evidence to link HHV-7 with human reproduction. It cannot be excluded that HHV-7 can be transmitted sexually, but the significance of this route, if any, is unknown at present.

### Concluding comments

HSV seroprevalence has been reported higher for a population of female fertility-clinic attendants compared to a population of pregnant women [61] and high HHV-6A/B antibody titers has been associated with a reduced chance of pregnancy [62]. Nevertheless, the collective set of data does not indicate an association between herpesviruses in semen and reduced fertility.

However, if sperm-associated herpesvirus is transmissible to the female cervix or perhaps even to the endometrium, as has been reported for HSV-2 [42], the consequences may be more difficult to estimate. For instance, the leukemia inhibitory factor (LIF) cytokine is essential for blastocyst implantation. Mouse LIF-null uteri are unreceptive, yet LIF-deleted blastocysts are unaffectedly implanted into normally functioning uteri [63]. Unexplained female infertility is not associated with mutations in the LIF gene [64]. Hu et al. [65] have shown that the production of LIF is partially regulated by p53 and that p53-null mice blastocyst implantation rate is significantly reduced. Herpesviruses such as HSV-1, HCMV, and HHV-6B have the ability to inactivate p53. Thus, implantation of the blastocyst could hypothetically be negatively influenced by viral infection of uterus tissue. Although the presence of HHV-6B in cells of the endometrium has not yet been investigated, it is possible that local infection or reactivation of these herpesviruses occurs and that viral activity in cells involved in endometrium receptivity would have a negative effect on human fertility.

Nevertheless, no evidence suggests so far that herpesvirus infection has a negative influence on reproduction. Indeed pre-pregnancy seropositivity to several viruses protects against primary infection during pregnancy, decreasing the risk for pregnancy complications such as preeclampsia [66]. Perhaps maternal infection by certain (herpes) viruses even protects against other more harmful infections.

The source of herpesviruses in semen has also yet to be determined. It is commonly known that mumps virus has the potential to infect the testis. Herpesviruses might share this ability to infect testis tissue. Indeed, EBV and HSV-1 and -2 have been isolated from human testis, and murine CMV (MCMV) from mouse testis. Additionally, HSV-2 and HCMV have been identified in prostate tissue, but the effect of herpesvirus infection at these sites is controversial [67].

Because of the risks of HCMV-mediated congenital defects, the interest for HCMV-screening on donor semen is increasing. Yet, it is not clear whether HCMV constitutes a threat to the semen recipient, but since HCMV isolated from semen is infectious [20,24,39], and since purification of HCMV positive semen does not efficiently eliminate the virus [22], the potential risk of HCMV transmission during assisted reproductive techniques (ART) cannot be ignored. Serology does not predict the presence of HCMV in semen or cervix [32], therefore screening of donor semen for HCMV DNA is necessary to prevent the use of HCMV-contaminated sperm in ART. Unfortunately, this would be costly, because shedding of herpesviruses fluctuates, necessitating screening of all semen samples rather than occasional checks [18].

## Abbreviations

AIDS: Acquired immunodeficiency syndrome; ART: Artificial reproductive technique; EBV: Epstein-Barr virus; FCA: Fertility-clinic attendants; HCMV: Human cytomegalovirus; HHV: Human herpesvirus; HSV: Herpes simplex virus; KS: Kaposi’s sarcoma; KSAV: Kaposi’s sarcoma-associated herpesvirus; PCR: Polymerase chain reaction; STD: Sexually transmitted disease; VZV: Varicella-zoster virus.

## Competing interests

The authors declare that they have no competing interests.

## Authors’ contributions

MDK surveyed literature and wrote the first draft of the article. PH organized the contents and submitted the article. Both authors read and approved the final manuscript.
